# Profiles of risk: a qualitative study of injecting drug users in Tehran, Iran

**DOI:** 10.1186/1477-7517-3-12

**Published:** 2006-03-18

**Authors:** Emran M Razzaghi, Afarin Rahimia Movaghar, Traci Craig Green, Kaveh Khoshnood

**Affiliations:** 1 Psychiatry, Tehran University of Medical Sciences, South Karegar Ave., Rouzbeh Hospital, Tehran, Iran; 2 Psychiatry, Iranian National Center for Addiction Studies, Tehran University of Medical Sciences, No. 669, South Karegar Ave., Tehran, 13366–16357, Iran; 3 Chronic Disease Epidemiology, Yale University School of Public Health, New Haven, 60 College St., CT 06520–8034, USA; 4 Epidemiology of Microbial Diseases, Yale University School of Public Health, New Haven, 60 College St., CT 06520–8034, USA; 5 Yale Center for Interdisciplinary Research on AIDS, Yale University, 40 Temple St. Suite 1B New Haven, CT 06510, USA

## Abstract

**Background:**

In Iran, there are an estimated 200,000 injecting drug users (IDUs). Injecting drug use is a relatively new phenomenon for this country, where opium smoking was the predominant form of drug use for hundreds of years. As in many countries experiencing a rise in injecting drug use, HIV/AIDS in Iran is associated with the injection of drugs, accounting for transmission of more than two-thirds of HIV infections. This study aimed to: describe the range of characteristics of IDUs in Tehran, Iran's capital city; 2) examine the injecting-related HIV risk behaviors of IDUs, and 3) suggest necessary interventions to prevent HIV transmission among IDUs and their families and sex partners.

**Methods:**

Using rapid assessment and response methods with a qualitative focus, six districts of Tehran were selected for study. A total of 81 key informants from different sectors and 154 IDUs were selected by purposeful, opportunistic and snowball sampling, then interviewed. Ethnographic observations were done for mapping and studying injecting-related HIV risk settings and behaviors. Modified content analysis methods were used to analyze the data and extract typologies of injecting drug users in Tehran.

**Results:**

Evidence of injecting drug use and drug-related harm was found in 5 of 6 study districts. Several profiles of IDUs were identified: depending on their socioeconomic status and degree of stability, IDUs employed different injecting behaviors and syringe hygiene practices. The prevalence of sharing injection instruments ranged from 30–100%. Varied magnitudes of risk were evident among the identified IDU typologies in terms of syringe disinfection methods, level of HIV awareness, and personal hygiene exhibited. At the time of research, there were no active HIV prevention programs in existence in Tehran.

**Conclusion:**

The recent rise of heroin injection in Iran is strongly associated with HIV risk. Sharing injection instruments is a common and complex behavior among Iranian IDUs. For each profile of IDU we identified, diverse and targeted interventions for decreasing sharing behavior and/or its harms are suggested. Some notable efforts to reduce the harm of injecting drug use in Iran have recently been accomplished, but further policies and action-oriented research for identification of effective preventive interventions are urgently needed.

## Background

In the Middle East, as it is worldwide, injecting drug use is a main route for transmitting HIV. The rise of injecting drug use, however, is still a relatively new phenomenon in Iran, where for centuries opium smoking was the predominant form of drug use [1-3]. There is a long history of opium use and production in Iran, though more recent times have witnessed the fall of opium production and the rise of Iran as a major drug transit country, due to its long border with the world's largest opium producer, Afghanistan. Consequently, increased availability and price fluctuations of heroin have led to an explosion of heroin dependency and, concomitantly, injecting drug use [1]. Factors associated with heroin injection in Iran and the neighboring countries have not been adequately studied.

Iran has the highest rate of heroin and opium addiction per capita in the world: 1 in 17 is a regular drug user and 20% of Iranians aged 15 to 60 is involved in drug abuse [4,5]. Of the government-estimated 1.8 million drug users in Iran, 9 to16 percent inject drugs, either as their primary or ancillary mode of drug use [1]. Thus, it could be roughly concluded that the injecting drug user (IDU) population in Iran is over 200,000. Other sources place the number of drug users in Iran at 3.3 million and the number of IDUs at over 300,000 [6-8]. Regardless of the actual number of IDUs, worrying trends suggest that, compared to non-injecting drug use, the prevalence of injecting drug use has increased more rapidly during the past decade and will continue to rise in Iran [2,9-11]. Indeed, many regard drugs as the most lethal threat to Iran today [5].

HIV/AIDS is closely associated with injecting drug use in Iran. Current data indicate that 67.3% of HIV positive cases and 85% of AIDS cases have a history of injecting drug use [12]. The number of HIV/AIDS cases is increasing rapidly in recent years, and estimates in 2004 indicate there are 30,000 people with HIV in Iran [13].

The few studies describing HIV risk in Iran underscore the main routes of transmission are sharing used injecting equipment, both inside and outside of prison [14]. In a study of 323 drug users with a past history of injection drug use, a history of syringe sharing and sharing at last injection was reported by 49.8% and 24.8% of respondents, respectively [1]. A recent seroprevalence study of attendees of three public drug treatment centers in Tehran found that a history of sharing syringes in prison was strongly associated (adjusted OR = 12.37 [95%CI: 2.94–51.97] with being HIV positive among the mostly male injectors. The prevalence of HIV among the IDUs in this study was 15.2% [15]. In a review of patient records from a drug treatment sample, more than two-thirds of the injecting drug users reported sharing syringes at some time, usually in a place other than their home or in prison[16].

Several studies have shown different patterns of risk-taking behaviors based on varying IDU demographic and other societal characteristics [17-19]. Social networks of IDUs play an important role in transmission dynamics and the success of prevention efforts [20-22]. Multiple studies have found differences between male and female IDUs on risky behaviors and the subsequent risk of HIV infection, underscoring the role of biological and social vulnerability factors. Research with IDUs in as geographically disparate locales as Marseille, France [18], Sydney, Australia [23,24], and Dublin, Ireland [25] has echoed the impacts of these vulnerabilities on drug use and HIV risk behaviors, and the implications of these disparities for future interventions. Age and drug experience also differentiate HIV risk, as has been shown in studies contrasting the risk behaviors of newly initiated IDUs with those of more experienced users [26,27]. In South-west China [28] and in 5 US cities [29] different ethnicities of IDUs have been found to be at differential risk. Significant variability in HIV risk, in fact, has been described at the neighborhood level even within zones of relatively uniform ethnic and sociodemographic composition [30].

Very little is known about the characteristics and risk profiles of IDUs in Iran. We sought to describe the range of characteristics of IDUs in Tehran, Iran, and to determine whether and how the harm reduction needs of IDUs in Iran differed within and among those at highest risk of HIV infection. We anticipated that our study would reveal some important differences in risk behavior and drug-related harm; hence we also aimed to describe these differences and to suggest specific HIV preventive interventions according to the IDU profile.

## Methods

### Approach

To accomplish our study aims, we drew from internationally accepted guidelines for rapid assessment and response methods, emphasizing a qualitative and ethnographic approach as the first step (The Rapid Assessment and Response Guide on Injection Drug Use (IDU-RAR)) [31]. This methodology was preferred for several reasons. Since drug use is an illicit act in Iran, enumeration for a random sample of drug users is difficult. Lacking objective, reliable data on the extent of drug use and risk behaviors there was little justification for a more complex and costly quantitative survey. Moreover, faced with constraints of limited resources, a qualitative study emphasis is more efficient for both understanding how risk behaviors are practiced and for generating hypotheses for future research and prevention initiatives. It is the spirit of the rapid assessment and response methods – embodied in the trademark elements of rapidity, triangulation, and induction–rather than the rigorous application of these methods, that propelled this qualitative research study.

### Site

The study was conducted in Tehran, the capital of Iran. Six districts with significant variability in cultural and historical background, social structure, extent of injecting drug use, and crime rates were selected according to secondary data from drug treatment clinics, police files, and discussions with experts such as Tehran police authorities and drug users, amassed from a previous WHO-sponsored study [1]. Sites were selected such that a contrast between three areas of high and three areas of low intensity of drug use and drug-related problems could be accomplished. We based this stratification on the primary indicator of drug-related arrests by district. At the time the study was initiated, there were no active HIV prevention programs in the study areas.

### Study population

The study populations consisted of key informants and drug users within the six districts. Using a purposive sampling method, a total of 81 key informants were selected, with the aim of capturing a wide spectrum of relevant community, health, and political perspectives. Key informants interviewed within each district included: the local sheriff, the local anti-drug police officer, physicians working in private or at public health centers, the director of the local health center, the local municipality agent at the district, the clergy of the local mosque, local pharmacists, the local blood transfusion bank officer, and the principals and teachers of girls' and boys' schools in the district. Concurrently, a total of 154 IDUs were interviewed in this study, sampled via several methods from a variety of settings within the districts including: convenience sampling from public drug treatment centers (N = 14), private drug treatment clinics (N = 5), and Narcotics Anonymous participants (N = 12); targeted sampling using ethnographic observations in public places (N = 71); and snowball sampling among out of treatment IDUs (N = 52), 15% (N = 23) of whom were female. Accessing and approaching female IDUs was extremely difficult, partly due to the male dominated drug scene and the stigma of drug use among women [16,32,33]. IDUs were eligible to be interviewed if they had injected drugs at least once in the 3 months prior to the study. Verbal informed consent was obtained from all participants. This study was reviewed by the institutional review board of the Iranian Welfare Organization as part of a WHO multi-site study.

### Data gathering methods and instruments

A combination of five data gathering methods were employed in this study, including secondary data gathering, in-depth individual interview, district-based focus group discussions, ethnographic observations, and mappings. Regarding secondary data, the available documents and data from relevant organizations (e.g., Tehran police, prisons department, legal medicine organization, drug control organization, blood transfusion organization) were reviewed. In-depth interviews with key informants focused on the broader situation of each district in general and IDU-related issues in specific within the district. Similarly, semi-structured and open-ended questionnaires were used in the in-depth interviews with IDUs to gauge their own drug use patterns, trends, and risk behaviors. To better understand the extent of drug use and risk behaviors at each district and the attitudes of IDUs toward injecting-based risk behaviors, related risk factors, and potential interventions, focus group discussions were held and co-moderated by fieldworkers from the Welfare Organization and ex-addicts. All in-depth interviews and focus groups were audiotaped and transcribed in Farsi. For more detailed information about the environment, ethnographic observations and mappings were done on the physical, geographic, social and cultural structure of each district. Ethnographers also captured observable characteristics of drug users, IDUs, and their risk environment, including, number and locations of shooting galleries, copping areas (i.e., the places where drug dealing occur), and the availability of health and social services. All fieldnotes were written for ease of synthesis with other data. IDUs were offered a small gift (e.g., wallet, watch) as remuneration for their participation in the study. Referrals to drug treatment services were also made as requested.

The fieldworkers who collected the interview and focus group data were either general practitioners or psychologists with a reasonable degree of experience in drug treatment. Moreover, as an attempt to build safe and empowering connections with drug users, one to two former IDUs were included in each district fieldwork team. These former IDUs were present during all interviews and focus group discussions with drug users and during ethnographic observations. In addition, at least one member of the fieldwork team was female. Selection of the former drug user fieldworkers was based on their communication skills, ability to respect full confidentiality regarding IDUs' personal information, and acceptable prior knowledge about the specific district. A training course of two working days was conducted for all the fieldworkers. The research team met weekly with the fieldworker teams to collect and review the data collected, to monitor targets met, and to deal with unanticipated situations.

### Analysis

All qualitative data collected underwent manual content analysis procedures, modified for realistic field application [31,34,35]. The resultant codings and subcodings for topics, concepts, and keywords, were applied to all the data and discussed by the research team (N = 6) and the leaders of each fieldworker team (N = 6) for consensus. Question by question, the research team and fieldworker leaders reviewed the data, analyzing and discussing the results according to the coding system collectively decided upon. Some of the responses for the questions were pre-coded according to the possible response options, while others were added as the content arose and the concept was operationalized [34]. Striving for consensus in interpretation, they also determined whether observed activities, risk behaviors, and drug use characteristics represented majority or minority trends, using all available district-level data. Preparation and written discussion of the research findings were based on this process and relied heavily upon the active involvement of and interpretation by fieldworker leaders, working in conjunction with the research team. It is from this iterative process that the typologies of IDUs presented herein were formulated.

## Results

Tehran, with an area of 2,000 km^2^, is the capital of Iran and its political and economic center. The population of Iran is more than 68 million, about one half of whom is under the age of 21 and 64% of whom dwell in an urban area. Tehran is home to about 17% of the Iranian population, or about 12 million citizens. The country's unemployment rate is estimated to be around 15%, and the literacy rate for men and women is 85% and 73%, respectively [36].

Data were collected from the fall of 2001 to the summer of 2002. The six study districts covered areas ranging between 0.4 and 8 km^2 ^with a total of 18.5 km^2 ^and had populations ranging between 2,000 and 286,000 with a total of more than 400,000 people. Some of the districts were primarily commercial trading areas, well-populated during the day but with a small nighttime population. Other districts were primarily residential areas, and some districts had a mix of both commercial trade and residential qualities.

Table 1 indicates the number of individuals interviewed by district.

**Table 1:** Number of interviewed individuals at each district

District name	No. of Key Informant Interviews on the general district situation, the extent of drug use and risk behaviors	No. of participants and FGDs* with IDUs on attitudes of IDUs towards risk behaviors, related factors and potential interventions No. participants (No. of FGD)	No. of participants and FGDs with IDUs on the extent of drug use and risk behaviors	No. of In-depth interviews with IDUs on their own drug use pattern, trends, and risk behavior
Maghsud-Beik (M)	13	0	0	0
Amiriye (A)	14	10 (1 FGD)	16 (2 FGDs)	8
13^th ^Aban (S)	13	11 (1 FGD)	17 (2 FGDs)	13
Bagh-e-Azari (B)	14	7 (1 FGD)	21 (2 FGDs)	14
Vali-e-Asr (V)	14	10 (1 FGD)	25 (3 FGDs)	10
Audlajan (O)	13	8 (1 FGD)	23 (3 FGDs)	14

Total**	81	46 (5 FGDs)	102 (12 FGDs)	59

* FGDs = Focus Group Discussions

** Some IDUs have participated in both FGDs and in-depth interviews.

Of the 154 IDUs who participated in the study, 18.7% were either illiterate or were barely able to read and write; 10.7% had graduated from high school or had higher education. Nearly one-third (32%) of IDU held a stable job; others were either unemployed, were involved in hustling, had illegitimate jobs (e.g., traded foreign currency), or engaged in illegal activities. About 18.2% of IDU in this study were homeless. The most frequently reported source of income for IDUs was the family. For the 23 women IDUs interviewed in this study, the most common source of income was through sex work. Most of these women were socially isolated, living alone or with another drug user.

Unless otherwise noted, findings were arrived at by triangulation of the key informant interviews, ethnographic observations and mappings, and focus group and in-depth interviews for each district. The findings on general characteristics of each district, extent of injecting drug use, characteristics of IDUs and their HIV risk behaviours are each summarized. While the extent of injecting drug use and HIV risk behaviors were similar in the 4 districts with a lower socioeconomic status, the 2 middle to high socioeconomic status districts differed substantially. Therefore, results are presented by the contextualizing factor of district socioeconomic status.

## District-level characteristics and drug use

### Higher socio-economic status: Maghsud-Beik district

*Maghsud-Beik *district is located in northern Tehran. In the past Maghsud-Beik was an old country side community once known for its villas and large gardens. Today, after increasing construction and commerce, Maghsud-Beik district is an integrated part of the metropolitan area of Tehran. The 2200 inhabitants of this district generally have a high socio-economic status, as evinced in their involvement in commercial occupations and/or higher levels of education. Aggressive and hostile behaviors in public are rarely observed. The police classify this district among the low drug use prevalence areas of Tehran. The extent of drug use is limited: drinking alcohol and, to a lesser extent, using opium on a traditional basis, are hobbies for a small number of elderly residents. Nevertheless, using hashish is not uncommon among adolescents.

Many interviewed key informants regarded the district as almost free of injecting drug use, which was subsequently confirmed by the lack of outpatient drug treatment attendees hailing from this district (i.e., from secondary data sources) and by ethnographic observations. This led to unexpected failure of fieldworkers to identify any IDUs for the in-depth interview and focus group discussion portions of this study. Most key informants believed the higher socio-economic status, greater family support, higher employment and law enforcement controls were powerful preventive factors against injecting drug use in the district. Notably, however, during the one month of ethnographic observation, there were no police anti-drug seizures in the district. Also, the local blood transfusion bank director estimated that about 70 IDUs come to donate blood each month, on the belief that a donation cleanses their blood. Without knowing how common an activity this is, it is hard to determine if blood bank attendance is capturing a large or small representation of the IDUs in the district. Nevertheless, assuming that only a portion of the IDUs who donate blood are residents of the district, it is not surprising that even snowball sampling techniques failed to generate a sample of IDUs for qualitative interviews.

### Middle socio-economic status: Amiriye district

*Amiriye *district is located in the central part of Tehran, with 70,000 residents but a commerce-driven fluctuating day and nighttime population. It is an historic, traditional district of Tehran, previously noted for its wealthy and religious inhabitants. The district's denizens are largely employed, with a majority involved in the iron market and motorbike industry and trade. Violent and illegal behaviors were uncommon in the district, according to key informants. Generally, the district is still an esteemed one and the police consider it to be clear of drugs. Despite this reputation, a significant number of IDUs admitted to the Welfare Organization drug treatment clinics have been residents of this district. Similarly, although some sources suggested that the district had no homeless drug users, fieldworkers observed cases of street drug users from nearby districts wandering about at nights. Used discarded needles were observed but only in some dilapidated parts of the district. The presence of injecting drug use in this district was contrary to our expectations, as the Amiriye district had originally been chosen as one of the sites with an expected low intensity of drug use. While the drug use may not have been as open, it was nonetheless present.

Several factors made identification of IDUs in this district a rather difficult task. The still strong family relations with drug users, the habit of using drugs indoors, and less frequent drug use by injection all challenged the research team's efforts to locate and interview IDUs. Recruitment through the major local drug distribution points was unsuccessful and few IDUs accepted to participate in interviews. The limited number and homogeneity in characteristics of identified IDUs, compounded by the failure to identify female IDUs, led fieldworkers to recruitment via more institutional routes (i.e., by checking files of Welfare Organization drug clinics). While these new efforts were met with greater success, neither homeless IDUs living within the district nor female IDUs were identified. Attempts to have some male IDUs recruit their female IDU friends were unsuccessful: the women rejected any cooperation for the sake of continued anonymity.

All IDUs from the Amiriye district who participated in the in-depth interview sessions were living with their families. Ethnographic observations and focus group discussions confirmed that most IDUs in the district were living with at least one family member, usually their mother. They appeared well-dressed and organized, with good personal hygiene and responded directly with relevant and reliable statements in their interviews. In descending order of frequency, IDUs were unemployed, car drivers, or unskilled workers.

In terms of injecting risk behaviors and the risk settings of injecting drug use, the IDUs in Amiriye district seem to be at relatively low risk. Injecting drug use is increasingly being practiced in private homes rather than public places, which was confirmed by our ethnographic observations. Using sterile syringes and disinfecting the spoon by boiling are common preventive practices among IDUs in Amiriye district. IDUs reported obtaining syringes from local pharmacies, which was later confirmed by two local pharmacists. Sharing of syringes was reportedly uncommon thanks to the adequate access to new, low-cost syringes, except in emergency cases. In the in-depth ethnographic interviews, only three of the eight IDUs reported to have shared injecting equipment in the two months prior to the study. One person had shared a spoon, one had shared a syringe only once while in prison and one had shared a syringe for injection three times. Their reasons for sharing injecting equipment revealed the barriers to safe injection: knowledge of safe injecting principles, imprisonment, drug craving and urgency to inject, and no access to clean syringes at time of injection. More specifically, one IDU felt confident in his actions because he had boiled the equipment, one was not concerned about the risk of shared injections, and one had been experiencing an urgent craving to inject drugs (i.e., withdrawal symptoms) while in prison and thus was not concerned with the risk of sharing injecting equipment at that moment.

### Lower socio-economic status: 13^th ^Aban, Bagh-e-Azari, Vali-e-Asr and Audlajan districts

Because the characteristics with respect to drug use, places of drug use, and HIV injecting risk behaviors for the lower socioeconomic status districts were similar, they are presented together, following a brief overview of each district's defining social, economic, and ethnographic features.

*13*^*th*^*Aban *district is located on the southern margin of Tehran and is home to about 31,500 people. Current inhabitants of the district are mainly former residents of slum areas around Tehran and migrants from rural areas, with a lower socio-economic status. Most inhabitants are relatives of retired workers from the industrial and agricultural sectors. Unemployment among young adults is high. The average education of the inhabitants is up to high-school grades. The family structure seems to be of an extended type. Gangs involved in robbery around Tehran reportedly live in this district and aggressive behavior and assault are obtrusive social problems. Furthermore, sexual violence and prostitution are commonly committed crimes. In rare instances, a family's main source of income is the wife's sex work. Some sex workers and drug users live in the ruins of an old, historic building at the corner of the district. The combination of public drug use and homelessness was noted by several key informants as a burgeoning social problem in this district.

*Bagh-e-Azari *district has 9000 inhabitants and is located in the south-east-center of Tehran. The district has a historical background and has many old houses. The current inhabitants of the district are generally of low socio-economic status, working as either peddlers or unskilled workers. Up to 90% of the district's inhabitants are migrants from other cities, a portion of who are Afghan refugees. Many inhabitants have faced unemployment recently. As a sign of the poor economic conditions, some families in the district cannot afford to rent a residential unit, so it is customary to reside in a single room. Many of the district's residents are relatives, and thus features of extended families still exist in the social structure. Crimes such as robbery, burglary and, blackmailing are not uncommon in the district. There has been an increase in the prevalence of sex workers during recent years, though resident sex workers prefer to conduct their work in other parts of Tehran. Homeless persons were seldom observed in the district, but were often seen in parks adjacent to the district. Although the police considered this area to be a problematic one for drug use, the Welfare Organization drug treatment clinics show no admittances from this district. Hence, this district was also originally considered to be one of low intensity injecting drug use.

*Vali-e-Asr *district is located on the outer rim in the south-west of Tehran and has a population of 286,000. The district was formerly known as a slum area. Current inhabitants are mainly of lower socio-economic status, reflected in the predominantly unskilled and semi-skilled employment profiles and the below average household income. Robberies and street fighting are frequent, as are other minor crimes such as smuggling of goods. Drug dealing is an established occupation of many inhabitants, including the youth. Law enforcement key informants evaluated the district as highly affected by drug problems. Finally, a large proportion of IDUs admitted to the Welfare Organization drug treatment clinics have come from this district.

*Audlajan *district is located in the east-center of Tehran situated adjacent to Tehran's great bazaar and is one of the oldest areas of the city. The district exhibits a daytime population flux due to its commercial nature to over 12,000 people which then falls to under 8000 at night. Nighttime residents are either unskilled workers at the bazaar and neighborhood industrial workplaces or unemployed individuals. Although merchants at the bazaar tend to be of higher socio-economic status and live outside of the district; permanent residents are generally of low socio-economic status. An increase in the number of immigrants in the district has led to a ghetto-ization based on ethnicity and cultural background. The typical Audlajan residence is set amid a narrow alleyway, in a section with older, architecturally historic one- or two-story houses consisting of two to four rooms on each side of a central yard. In each room, three to four individuals, mostly single males, live together. The yard or the roof is rented overnight to non-residents, both male and female. The major social problem in Audlajan is homelessness among both men and women. The research team encountered homeless women in the district whose husbands were in prison. Criminal activities such as business scams, reselling and street-based selling of merchandise, robbery, and pick- pocketing occur often in the district. More disturbingly, there were reports of the presence of gangs that resettle runaway girls into prostitution and drug trafficking networks in the district. According to police, drug use is highly prevalent among residents of the district.

### Characteristics of injecting drug users in lower socioeconomic districts

Drug use in various forms, including injecting, is almost epidemic in all of the lower socio-economic status districts. Contrary to our expectations, even the Bagh-e-Azari district which had reportedly no residents admitted to drug treatment, had a sizable injecting drug use problem. In all four districts, a majority of the IDUs participating in the in-depth interviews and focus group discussions lived with their families and only a small group of IDUs was homeless. Regarding the employment status of IDUs, in one district they were more skilled workers, in another unskilled workers and in the other two districts most drug users were unemployed, had temporary occupations or afforded their drug expenses by criminal involvement such as drug dealing. Among one district's homeless IDUs there were even some individuals with a higher education from a wealthy family background. In ethnographic observations from public places, most of the IDUs there were homeless males in poor health. Ethnographic observations revealed that these young male IDUs had multiple social problems (poverty, unemployment, divorce, homelessness, familial conflict, etc.), lived in public parks or other opportunistic locations (e.g., ruined buildings), and had poor hygiene and prominent tooth decay. Finding female drug users was difficult during the daytime in these four districts, but most of the female IDUs seen wandering the streets in the evenings had come to purchase drugs and were thus not district residents.

### Places for injecting drug use in lower socioeconomic districts

According to key informants and IDUs in all four districts, injecting drug use takes place mostly in public places such as parks, gardens, ruined buildings, canals and bridges, public bathrooms, streets and alleys. Using ethnographic observations, injecting practices were observed in various public places. Fieldworkers witnessed group injection and syringe sharing upon multiple occasions. Direct observation revealed that the places commonly used for injecting drugs were in poor environmental health condition. In one district, an ethnographer reported: "Adjacent to the district an unutilized land is used as a shooting gallery by IDUs. Groups of IDUs inject drugs poured from a common cup. After injection they wrap their needles in a tissue and put it in a corner for the next use. Many of the individuals sleep at the same place, too." As one may expect in areas with high public drug consumption, ethnographers observed streets and alley corners littered with used syringes, lampblacks (a gas cooking device), spoons, cigarette filters, and blood-stained clothes. In one district, schools' principals expressed their unhappiness at learning that some school children were already exposed to scenes of injecting drug use in the streets on their way to school. This district appeared to be functioning as a safe haven for street drug users from other parts of Tehran and other cities, and offered ready access to drugs. In ethnographic observations from one of the districts, no cases of injecting drug use practice were observed in streets and alleys, but they were recorded in abandoned and ruined buildings.

Among the private places, cardinal places for group drug injecting were "safe houses", where private houses belonging to people who lived alone would serve as a place for a group of close friends to inject together. For one district specifically, and in all districts more generally, these private houses were pinpointed as safe places for injecting drug use. No charges for the use of the space were levied nor were needles sold in these houses; they were not "shooting galleries" but safe havens.

### HIV risk behaviors: Injecting conditions and sharing practices in lower socioeconomic districts

In focus group discussions, participant IDUs from the four districts stated that as pharmacies have become more inclined to provide sterile syringes, they do not hesitate to use new sterile injecting equipment. While it is unclear why or how this change in attitude among pharmacies selling syringes took place, the resultant more accessible source of clean syringes is noteworthy. IDUs have become increasingly eager to use sterile syringes but a majority still reported using unsterile syringes. Most IDUs try to disinfect injection equipment by boiling the syringes or heating the sharp over a direct flame, a common practice noted when injecting took place at a home. Drug users who inject in public places, on the other hand, continued to use unsterile syringes. Their syringe hygiene habit was to use saliva (by licking the syringe) and plain water or to clean the syringe with a cloth or paper. A few street drug users admitted to not cleaning their injecting equipment at all or just with their hand. After injecting, the disinfecting habits of street drug users were to wash the injecting equipment with water and wrap it in a plastic sheet so that it was ready for the next use.

Half of the IDUs in the four districts had a history of daily or more frequent sharing of injection equipment during the one or two months prior to the in-depth interview. Female and homeless IDUs reported that sharing syringes is typical, though other IDUs did not concur. A homeless IDU in one district stated: "It was frequent that a group of two or three IDUs share a single needle three to four times a day for a period of two months, before spending some money for buying another needle." Although rare, renting injection equipment occurs, but sharing on a friendship basis is more the habit. The sharing of other drug preparation equipment such as the spoon or cooker is more common than sharing the syringe itself. Indeed, IDUs indicated that in all group injections a single cooker is used.

A small minority of 'end-stage' IDUs, those who were incapacitated by their addiction and whose health status had deteriorated to near-death, were totally dependent on using publicly discarded syringes such as those left at street corners. They usually washed the syringes with plain water and, prior to injection, some habitually drew the needle over their tongue in order to disinfect it. There were some reports of these end-stage IDUs, as well as other homeless individuals, collecting used syringes and, after washing them with plain water, peddling them to other IDUs.

## Typologies of injecting drug use

Table 2 overviews the typologies of injecting drug use formulated from a synthesis of the study findings by socioeconomic status. We focused on three domains in our typology: stability, syringe hygiene, and syringe sharing behaviors. Stability refers to the degree of order in one's life and drug using circumstances, including their job, family setting, and choice of injecting location. For example, a stable situation is characterized by living at home, marriage, employment that is not illegal, and injecting at home, whereas an unstable situation is characterized by homelessness, not being married, illegal employment, and injecting in public places. We conceptualized stability as a continuum, and operationalized it by the social, environmental, economic and other relevant characteristics describing IDUs in each typology. Syringe hygiene refers to the practices employed by IDUs to clean their syringes, injecting equipment and the site of injection. Syringe sharing behaviors are those behaviors practiced by IDUs that include direct sharing of injecting equipment as well as syringe-mediated sharing behaviors, which involve the use of syringes to divide drug solutions [37,38]. The syringe hygiene and sharing behaviors generally fall along a continuum of risk for injecting-related harms including blood-borne and injection-site infections. Given that we have 3 main typologies of IDUs, the continuums are theoretical, not specific.

**Table 2:** Profiles of injecting drug users, their injecting risk behaviors*.

	**Private, stable injectors**	**Unstable injectors**	
Primary districts representing the profile	A	S, B	V, O

Relative size †	Majority	minority	minority

**Stability continuum**	Unstable -------------------------------------------------------- Stable		

Distinguishing characteristics	Predominantly male, live at home with family, stable resources (shelter, food, income from family),	Predominantly young males, live at home with family but inject in public places or 'secure houses', limited resources (unemployed, dependent on family/ friends, some criminal involvement), shared injections for economic and emotional support, prevention of overdose	Predominantly young males, live and inject in public places, poor health & hygiene, very limited/no resources (unemployed, criminal involvement common), multiple social problems (divorce, poverty, familial conflict); the most extreme of this group were the 'end-stage users'
HIV & injecting risk continuum	Higher risk ---------------------------------------------------- Lower risk		

**Syringe use practices**: hygiene and injecting customs	Boiling, direct heating of point	Licking point, rinsing with water, flushing with boiling water, wiping with cloth or paper; repeated reuse of syringes; injecting practices involving repeated injection of blood ("blood play")	Licking point, rinsing with water, wiping with cloth or paper, or none; repeated reuse of syringes; injecting practices involving repeated injection of blood ("blood play"); often inject alone
**Syringe use practices: **sharing behaviors	Few syringe sharing occasions; frequent other equipment sharing	Many sharing occasions; sharing of other equipment typical	Primarily syringe sharing occasions; primarily sharing of other equipment
Access to harm reduction materials #38; risk awareness	access to pharmacy-sold syringes	limited access to pharmacy-sold syringes; aware of HIV and injecting risks but continue to share	limited access to pharmacy-sold syringes and lack of awareness of risk drives sharing behaviors

*Note, Maghsud-Beik district was not noted for its injecting drug use and thus does not appear as a primary district for any of the IDU profiles.

A = *Amiriye*, S = *13*^*th*^*Aban*, B = *Bagh-e-Azari*, V = *Vali-e-Asr*, O = *Audlajan*

† Relative size was determined through the key informant interviews and secondary data sources for all districts (e.g., admissions to drug treatment, overdose deaths, etc.)

IDUs in the six districts can be divided into two groups: a majority who lives with their families and inject at home and a minority who inject in public either because they do not want or cannot inject at home or because they are homeless and experience a high degree of deprivation. Injecting-related HIV risk behaviors differ for these two groups. Syringe sharing, while a common habit among the publicly injecting and homeless IDUs, is seldom practiced among IDUs in the larger group. Almost all IDUs living with their families obtained syringes from pharmacies. This primary syringe source indicates good accessibility to new, low-cost syringes, availability of the syringes to those who need and can afford them, and high acceptability (i.e., less stigmatization) of purchasing syringes from a community pharmacy. In instances of reuse or sharing of a syringe, IDUs living with their families who injected at home preferred to boil the syringe in advance of injection to disinfect it. They believed that sharing injections should be avoided because of the assumed risk of HIV and hepatitis infection from this practice. While there is laboratory-based evidence to support that HIV-1 viability in syringes is reduced by boiling and subjection of syringe to heat, this method of harm reduction is inconsistent and far inferior to the use of a new syringe [39]. These syringe use practices may be interpreted as a positive sign that the group of IDUs injecting at home and living with their families recognizes the value of self-care, hygiene and is relatively educated about injecting-related risks.

The other group comprises a minority of the IDUs but they are at much higher risk for harms related to injecting drug use. This group seems to be less careful with their personal hygiene and overall health, they also do not hesitate to use drugs in public places, commonly share syringes, practice unsafe methods of injection, and commit illegal acts for gaining money and drugs. Despite their similarity in HIV and injecting risk practices, the unstable injectors are not a homogeneous group (see Table 2). A larger subgroup seems to be concerned about the harms related to unsafe injection and wishes to be able to quit injecting drugs. According to them, more money or access to more pure drugs would allow them to stop injecting and return to smoking drugs. Earlier studies among drug users in Iran had found that, compared to the more traditional method of smoking drugs, injecting is a highly stigmatized and less preferred route of drug use [1]. Yet these IDUs were apathetic about the hazards of sharing syringes in spite of their knowledge of the consequences of sharing equipment.

On the farthest end of the stability and risk spectrum, a small group of homeless IDUs are at extremely low levels of health status and hygiene and greatest marginalization. This group suffers from multiple abscesses, lives in a dire, filthy state, and is apathetic about their own life and their environment unless seeking drugs. Many of these IDUs even sold or lent their national identification cards, their primary personal identification, to others in return for drugs. The most extreme case, and furthest to the right of the Table 2 spectrum, is the 'end-stage' user, whose health status has deteriorated to near death. Preventive interventions such as short-term risk reduction training and HIV/hepatitis awareness programs and even syringe exchange programs seem to be of little or no value for this highly unstable group.

## Discussion

This study was able to locate IDUs in five of the six districts of Tehran examined. We were unable to contrast the HIV risk behaviors in areas with low drug use to those with high drug use, as we observed injecting drug use in nearly all of the districts, contrary to our expectations. Synthesizing data from multiple quantitative and qualitative sources, we identified and described the unique patterns of injecting drug use in Tehran. The findings from this study provide some insight about the profiles of IDUs in relation to their social context and their HIV risk behaviors but they also suggest needed typology-targeted interventions. Table 3 overviews some typology-suggested interventions.

**Table 3:** Typology-targeted interventions

	**Private, stable injectors**	**Unstable injectors**	
Suggested interventions	Syringe and injecting hygiene awareness and training; improve access to pharmacy-sold syringes	Social support, training, increased access to low-threshold methadone maintenance treatment, syringe exchange programs, improve access to pharmacy-sold syringes; safer injection facilities	Mass syringe distribution and recollections, outreach, low threshold services (counseling, social support); safer injection facilities

The typology and profile of risk of the private, stable injectors suggests that further efforts to increase awareness and training about the harms associated with reuse of syringes or even appropriate methods for disinfection of used syringes would be both feasible and of particular harm reduction benefit. Since this group makes up the majority of IDUs, sophisticated preventive approaches are suggested. In populations where IDUs are part of a relatively stable social network, sustain steady partnerships and friendships, and confine their sharing of injecting equipment to those persons they know well, HIV has less of an opportunity to reach devastatingly high prevalence rates [21,40,41]. It has been asserted that prevention strategies that strengthen this social network and make use of it for educating IDUs, especially new IDUs, will be more successful than strategies based on approaching individuals without taking the social environment into account [20,21,41]. Furthermore, this group of IDUs appears to be more eager to seek treatment, albeit with high relapse rate, so motivation to change risk behaviors could produce greater reduction in injecting-related harms than in less motivated IDUs. Another potential intervention worth exploring for Iranian IDUs arose from the ethnographic field work: blood banks could serve as an additional place for communicating blood borne virus prevention education and improved injecting hygiene to drug users who visit these establishments for "blood cleansing".

Interventions suggested for the less stable injectors take a different form. The first subgroup of unstable injectors (middle column of Table 3), although at high risk of experiencing the harms of unsafe injection, presents a potential focus of harm minimization programs, especially social support, risk reduction skills training, coverage by opioid substitution maintenance treatment programs, bleach and sterile syringe providing programs, and safer injection facilities. For the second subgroup of unstable injectors (right column of Table 3) who live in small groups, perhaps one harm reduction response could be peer-based delivery of free sterile syringes with a scheduled recollection of the used syringes. This service could be offered with concomitant social support measures on a short-term basis. The approach could establish a rapport with the IDUs and also help to bring their injecting habits under observation. Regardless of the living situation of these highly unstable IDUs, the involvement of peer drug users in outreach programs to deliver such services might be more acceptable to this typology of IDUs and more cost-effective for the community. Safer injection facilities could also connect IDUs to harm reduction and additional support services, while providing clean injecting materials and a safer place to inject.

We elaborated on the contextualizing factors of socioeconomic status and other district-level characteristics, as they are known to influence drug using behaviors and HIV risk [19,42]. For example, the degree of criminal activity in a district tends to increase police presence, which in turn raises the fear of arrest in drug users and promotes more covert, more efficient routes of drug administration. In these settings, injection of drugs is more common and failing to take precautions against the risk of infection are of less immediate consequence, compared to the risk of arrest and prosecution. In another example, the lack of economic development and the presence of abandoned and ruins of buildings in a district permits multiple areas for public drug consumption, especially for those who cannot use drugs at home (e.g., are homeless) or who choose not to use at home (e.g., lives with family who may or may not know they use drugs). Such scenarios broaden the discussion of the profiles of HIV risk, and argue for consideration of interventions like community revitalization, healthy urban planning, and crime prevention programs as part of HIV risk reduction. A recent study by the Economic Intelligence Unit assessing living conditions in 127 cities around the world by looking at 40 indicators of stability, healthcare, culture and environment, education, and infrastructure ranked Tehran among the ten least livable cities [43]. These data suggest that an improvement in social conditions would benefit more than just the reduction of HIV risk.

The social, cultural, and economic diversity in the many regions and districts, compounded by the different type and prevalence of social problems including drug use observed in this study, suggests that designing and implementing a general harm reduction program with applicability to all areas would not be fruitful. As formal institutions seem to be unable to respond with such specificity, future facilitation of involvement and participation of non-governmental organizations (NGOs) and the traditional informal social institutions is needed. One superb example in Iran is the work of the Iranian non-profit organization Persepolis. They have succeeded in piloting a methadone maintenance program, establishing an outreach program, and spearheading a needle exchange to meet the needs of IDU which have otherwise remained unmet [44,45]. Unfortunately, their coverage is limited to a small section of Tehran.

Because drug-user risk patterns are manifested locally, at the level of individuals and small networks within a specific microsocial context, there is a critical need for research methods that permit effective identification, systematic description, and detailed comparison and analysis of local drug-using populations, risk behaviors, and social influences on injection patterns. This type of highly contextual research [35,46] allows the development of "grounded prevention efforts" that are specifically targeted toward empirically- verified features and determinants of actual risk in given social environments. The research that propelled the Australian and Canadian pilot safer injection facilities are excellent and timely examples of this "bottom-up" approach [47-49]. It is the hope that our findings may inspire additional research and discussion of targeted harm reduction responses, such as a safer injection facility, which emerged as an appropriate potential and needed intervention for one or more of the drug user typologies.

The current drug policy responses in Iran depart from the anti-trafficking focus and draconian measures of the past, and give an indication of the plausibility of implementing the typology-suggested interventions and a more public health approach to addiction. The government is embracing a harm reduction response to the epidemic of injecting drug use [50]. The progressive efforts to address many of the social and health effects of drug use in Iran are being driven by leadership that appears to grasp the reality and enormity of the domestic drug use problem, and the potential for an injecting-driven HIV epidemic in Iran. Over the past 6 to 8 years, their efforts are notable: the expansion of therapeutic communities, Narcotics Anonymous, and outpatient clinics; sponsoring pilot substitution treatment programs (methadone and buprenorphine) and support of their expansion in principle and in action (in Spring of 2005, the parliament voted to allow any doctor in Iran to dispense methadone, under strict monitoring guidelines); implementation of outreach programs and enlarging the network of existing outreach mechanisms, such as the more than sixty "Triangular Clinics" that are devoted to the health concerns of high-risk individuals like sex workers and drug users; support of needle exchange and pharmacy-sold syringes programs to operate and vend syringes legally to drug users (see January 2005 decree of the judicial branch of the Islamic Republic of Iran supporting needle exchange and warning against interference with these 'needed and fruitful' public health interventions) [3]; piloting prison-based NEP programs; and liberalizing drug-sentencing guidelines (see Justice Minister Ayatollah Mohammad Esmail Shoshtari's letter to prosecutors to ignore the current laws on the books and to defer to Iran's Health Ministry to counter the spread of AIDS and hepatitis C) [50]; and subsidizing the cost of treatment for substitution therapies for drug users as well as antiretroviral therapy for those who are HIV positive. The openness to many of these individual and social structural responses indicate that there is a unique window of opportunity for remarkable reduction in drug-related harm in Iran, provided that the momentum can be maintained and that rigorous evaluations are undertaken to objectively gauge effectiveness.

There are notable limitations to this study. First, the study was conducted in only six districts in Tehran, so generalizations of analyses and results to the whole social structure of Tehran and to other districts in the metropolitan area are limited. Second, drug use and injecting drug use in particular are illegal, thus individuals involved in the study belong to hidden populations, which inherently compromise the researcher's ability to construct a random sample. We did not intend to conduct inferential analyses or to generalize beyond the scope of this study population so this limitation is partly attenuated. Our purposes were descriptive and qualitative in nature, highlighting the aspects of IDUs' HIV risk most relevant to further research and exploration of harm reduction initiatives. Third, this study did not focus on some important aspects of drug use at the individual level (e.g., psychiatric comorbidities) nor did we examine the structural impediments that increase injecting-related risk (e.g., policing practices) in the study districts [51]. Specific research studies should be conducted to determine if a more specific harm minimization response is warranted for special populations, and to explore the role of structural effects in the formation of IDU typologies and in intervention success. Finally, our study relied heavily upon self-report data, which is open to selection and information biases. For example, the inability to find IDUs in the Maghsud-Beik district could have been due to the fieldworker team employed therein and the effects of social desirability bias on reporting drug use in this higher socioeconomic district. Nevertheless, the inclusion of complementing secondary data source reviews and ethnographic observation and mapping data help to improve the study's conclusion validity.

In conclusion, heroin injection is commonly practiced among drug users in Iran. There are several distinct subgroups of Iranian IDUs, among whom sharing injecting instruments is a common and complex behavior. For each profile of injecting drug use and taking into account the unique social context, a specific approach for reducing injecting-related harm and HIV risk behaviors should be applied. Despite notable recent efforts, action-oriented research for identification of effective preventive interventions for IDUs in Iran is urgently needed.

## List of abbreviations

AIDS = Acquired immunodeficiency syndrome

HIV = human immunodeficiency virus

IDU = injecting drug user

NGO = non-governmental organization

WHO = World Health Organization

UNODC = United Nations office on Drug and Crimes

FGDs = Focus Group Discussions

## Competing interests

The author(s) declare that they have no competing interests.

## Contribution of authors

ER conceived of the study, oversaw the data collection, led the data analysis, and drafted the manuscript. AM assisted in study design, data collection, and data analysis. TG participated in data analysis and interpretation, and drafting the manuscript. KK advised the data interpretation and manuscript preparation. All authors read and approved the final manuscript.
